# Double-Layered Polymer Microcapsule Containing Non-Flammable Agent for Initial Fire Suppression

**DOI:** 10.3390/ma15217831

**Published:** 2022-11-06

**Authors:** Dong Hun Lee, Soonhyun Kwon, Young Eun Kim, Na Yeon Kim, Ji Bong Joo

**Affiliations:** Department of Chemical Engineering, Konkuk University, Gwangjin-gu, Seoul 05029, Korea

**Keywords:** microcapsules, double-layered, initial fire suppression, electrical fire

## Abstract

Fire in energy storage systems, such as lithium-ion batteries, has been raised as a serious concern due to the difficulty of suppressing it. Fluorine-based non-flammable agents used as internal substances leaked through the fine pores of the polymer outer shell, leading to a degradation of fire extinguishing performance. To improve the durability of the fire suppression microcapsules and the stability of the ouster shell, a complex coacervation was used, which could be microencapsulated at a lower temperature, and the polymer shell was coated with urea-formaldehyde (UF) resin. The outermost UF resin formed elaborate bonds with the gelatin-based shell, and thus, the structure of the outer shell became denser, thereby improving the loss resistance of the inner substance and thermal stability. The double-layered microcapsules had an average particle diameter of about 309 μm, and a stable outer shell formed with a mass loss of 0.005% during long-term storage for 100 days. This study confirmed that the double-layered microcapsules significantly improved thermal stability, resistance to core material loss, core material content and fire suppression performance compared to single wall microcapsules. These results indicated that the double-layered structure was suitable for the production of microcapsules for initial fire suppression, including highly volatile non-flammable agents with a low boiling point.

## 1. Introduction

Electrical fire accidents have been caused by short circuits due to overload in a domestic electrical outlet or distribution boards in recent years, and the scope of these fire accidents has been expanded by increasing the usage of energy storage systems (ESS) for alternative energy sources, such as photovoltaic power generation and nuclear power generation. It has been reported that more than 10% of nuclear power plant fire accidents were caused by electrical factors [1], and 73.08% of ESS accidents were caused by electrical factors at photovoltaic power generation plants [2]. Electrical fire accidents generally occur in enclosed spaces, such as an electrical distribution panel box, making it difficult to suppress and respond to initial fires. In order to deal with this type of fire accident, the main system was installed, which stores the extinguishing agents in a container, detects fires through fire detection systems and releases fire extinguishing agents. However, it has disadvantages such as high installation/maintenance costs and malfunctioning automatic fire detection systems. In addition, in the case of fires of lithium-ion batteries, it is extremely difficult to extinguish them by conventional methods due to the thermal instability of the batteries [3].

Such electrical fires should be suppressed with a fire extinguishing agent with flame retardant properties, such as halide agents (halon series). However, several agents, such as halon 1301, halon 2402 and halon 1211, cause a significant problem of residual substances generated after fire suppression, which destroy the ozone layer in the stratosphere. Thus, they were completely banned under the Montreal Protocol (1989) and the Kyoto Protocol (2005). Recently, fire extinguishing agents have been replaced by eco-friendly materials, such as HFC-227ea and FK-5-1-1-12, which have a low global warming potential (GWP) and an ozone depletion potential close to almost 0. However, these eco-friendly fire extinguishing agents are difficult to store and utilize due to the low vaporization temperature and high vapor pressure [4,5,6]. In order to overcome these disadvantages, methods of using an aerosol system [7] and encapsulating the fire extinguishing agent with a polymeric material have been proposed [8,9,10,11,12,13]. In particular, microencapsulation of fire extinguishing agents has been studied recently for the prevention and suppression of fire in lithium-ion batteries and similar energy storage systems. It is well known that the thermal instability of lithium-ion batteries is generally caused by a failure of the cell separator. It is difficult to fully extinguish fires with conventional smothering extinguishment [3]. On the other hand, since an eco-friendly halide fire extinguishing agent such as Novec-1230 has a high vaporization enthalpy, it can easily cool down as well as smother extinguishment. Thus, a microcapsule containing this type of liquid agent is suitable for an electrical fire in energy storage systems.

So far, microencapsulation technology has been used mainly in food and medical applications. Recently, microencapsulation technology has been applied to various practical applications. Phase change materials (PCMs) have been used as core materials for microcapsules and applied to energy storage and conversion [14,15,16,17,18,19,20]. In addition, some researchers have proposed microencapsulation-based fire extinguishing devices. The microencapsulation techniques are mainly divided into two categories according to the outer wall materials, such as polymer microcapsules and inorganic microcapsules. Inorganic microcapsules cannot be applied as fire extinguishing microcapsules in case of electrical fire accidents, as the conductivity of the shell materials is not suitable. In addition, there are still challenges to mass-producing microcapsules containing fire extinguishing agents as internal core materials due to their intrinsic volatile properties, which cause difficulties in the manufacturing process. 

Some pioneering researchers have suggested an efficient microencapsulation technique for highly volatile core materials using polymer shell materials. Representative methods for polymer microcapsules include *in situ* polymerization, interfacial polymerization, UV polymerization and complex coacervation. Vilesov et al. [8] fabricated microcapsules, which contained Novec-1230 with photo gelatin by the coacervation method. They synthesized coacervation at a lower temperature than other methods and were able to easily encapsulate core materials by simply adjusting the pH value [14,16,17]. However, microcapsules with polymer shells have some weaknesses, such as lower mechanical strength and stability toward loss of internal substances compared to inorganic microcapsules [20]. The loss of core materials leads to a decrease in fire suppression effectiveness with prolonged use. To improve the stability of the core substance in the polymer microcapsule, researchers introduced mineral materials into the polymer matrix of the shell [21,22]. Vilesov et al. [9] improved core stability and resistance to loss of internal materials by dispersing montmorillonite in an aqueous gelatin solution to form the outer shell. Yim et al. [12] used Novec-7300, a similar non-flammable liquid agent, instead of Novec-1230, and synthesized a temperature-responsive microcapsule that could be used in a lithium-ion battery by using polymethyl methacrylate (PMMA) as the outer wall material. Zhang et al. [13] reported microcapsules reacting to lithium-ion battery fire using melamine urea formaldehyde resin as shell material. The above-described methods had complicated procedures to prepare the prepolymer for forming the shell, especially PMMA characterized by poor mechanical strength, and required additional materials to reinforce mechanical strength [20]. As mentioned, the microcapsule procedure for fire extinguishing agents is still complex, and minimizing the loss of internal core substances is still a major challenge.

In this study, we fabricated microcapsules by the coacervation method with gelatin and sodium hexametaphosphate to improve thermal stability and resistance to loss of core materials and coated microcapsules with urea-formaldehyde resin to form double-layered microcapsules. The effect of the double-layered structure on fire suppression was investigated by means of characterization and fire suppression test. The purpose of this study is to propose an effective alternative solution to an electrical fire accident from an energy storage system or other electric facilities by initial fire suppression using double-layered microcapsules.

## 2. Materials and Methods

### 2.1. Materials

Gelatin from bovine skins (type B, bloom ~225 g) was purchased from Sigma-Aldrich (St. Louis, MO, USA), and sodium hexametaphosphate was purchased from Daejung Chemicals & Metals Co., Ltd. (Gyeong-gi, Korea) and used as shell materials. Methoxynonafluorobutane (MNFB, Sigma-Aldrich, Burlington, MA, USA) was us ed as core material. The physicochemical properties of MNFB are shown in Table 1. Urea (Junsei Co., Tokyo, Japan) and formaldehyde (37%, Daejung Chemical & Metals Co., Ltd., Gyeonggi-do, Korea) were used as coating materials. Polyvinylpyrrolidone (K30, Daejung Chemical & Metals Co., Ltd., Gyeonggi-do, Korea) was used as an emulsifier. Glutaric aldehyde (50 wt%) was purchased from Daejung Chemicals & Metals Co., Ltd. and used as a crosslinker. Hydrochloric acid solutions (0.1 M and 1 M) were purchased from Daejung Chemical & Metals Co., Ltd. and used to adjust the pH value of the solution.

### 2.2. Preparation of Microcapsule

**Figure 1:**
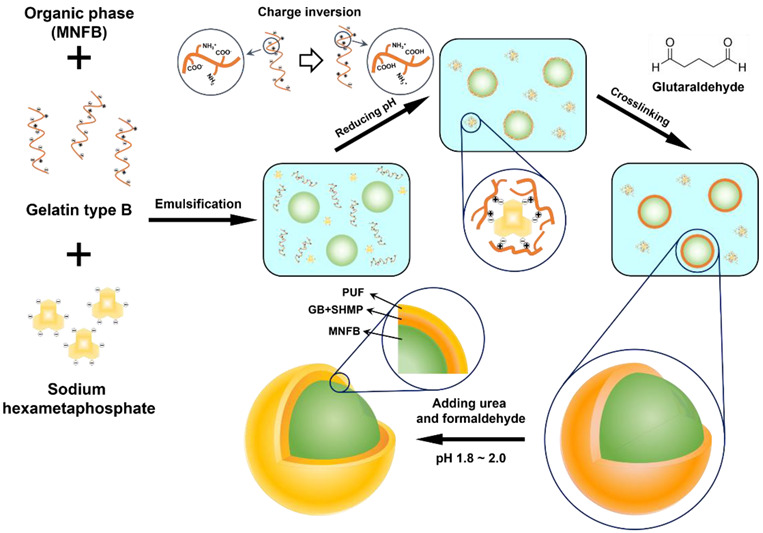
Schematic illustration of the DL-MC microencapsulation process.

A single-layered microcapsule containing the non-flammable agent, methoxynonafluorobutane (MNFB), was fabricated by the complex coacervation of type B gelatin (GB) and sodium hexametaphosphate (SHMP). Double-layered microcapsule was prepared by additional coating with urea-formaldehyde resin by in situ polymerization. The process of microencapsulation is shown in Figure 1. The process was carried out in 4 steps: (1) an oil/water (O/W) emulsion formation step, (2) a complex coacervation step, (3) a cross-linking step for shell hardening and (4) an outer shell coating step. 

In the O/W emulsion formation step, MNFB was used as the oil phase, and the GB-SHMP mixture was used as the aqueous phase. GB (4.5 g) was swelled in 100 mL deionized water for 30 min at room temperature, and SHMP (0.3 g) was dissolved in deionized water (50 mL) and heated to 70 °C for 1 h. The GB-SHMP mixture was cooled to 30 °C, and MNFB (17 mL) was added to the mixture. The O/W emulsion was attained after vigorous stirring for 5 min, and the temperature of the emulsion was kept at 35 °C. 

Aqueous HCl solution (0.1 M) was then slowly added with gentle stirring. As shown in Figure S1, tiny coacervate gels were formed as the pH value decreased. The agglomerated coacervate gels were sufficiently distributed over the surface of the oil emulsion by maintaining the pH value at 4.8 for 15 min. The pH value was then slowly lowered to 4.45, and a stable polymer shell was formed by sorption of another coacervate gel. 

Before cross-linking, the obtained mixture was cooled to 5 °C for 2 h and maintained at this temperature for 4 h to ensure sufficient mechanical strength of the polymer shell. Subsequently, glutaric aldehyde (5 mL, 25 wt%) was added at a rate of 0.1 mL/min using a syringe pump, and the cross-linking was preceded by raising the temperature to 30 °C. After 12 h, single-layered microcapsules (MCs) were obtained by decanting and drying at room temperature. 

MCs (10 g) were slowly added to deionized water (150 mL), and polyvinylpyrrolidone (PVP, 0.5 g) was added to avoid agglomeration of the microcapsules under conditions of vigorous stirring for 15 min. Urea (1.875 g) was added into the solution, and the solution was heated to 35 °C. After the urea was completely dissolved, the pH value was lowered to ca. 1.8 by HCl solution (1 M). Subsequently, formaldehyde (4.16 mL) was added, and the mixture was stirred for 1.5 h to form a second layer. Double-layered microcapsules (DL-MCs) were obtained by decanting after cooling and dried for 24 h at room temperature.

### 2.3. Characteristics

We characterized the particle size, shell thickness and morphology of DL-MCs by using a particle size analyzer (Sympatec GmbH, Clausthal-Zellerfeld, Germany) and field emission scanning electron microscopy (FE-SEM, SU8010, Hitachi, Japan). FTIR spectroscopy (FT/IR-4100, JASCO, Tokyo, Japan) was used to characterize the functional groups on the shell of the microcapsules. The presence of the core materials was confirmed by ^19^F-nuclear magnetic resonance spectroscopy (^19^F-NMR, JNM-ECZ500R/S1, Jeol, Tokyo, Japan) analysis. Thermal properties were characterized by thermogravimetric analysis (TGA, TGA-N-1000, Scinco, Korea). The samples for TGA analyses were exposed to ambient conditions for more than 100 days. TGA analysis was carried out under an air atmosphere, and the sample was heated from 30 to 350 °C at a heating rate of 10 °C/min.

### 2.4. Fire Suppression Test

The fire suppression test was conducted in a transparent container (20 cm × 20 cm × 30 cm) with 2 holes in the side wall to prevent the natural smothering extinguishment, and n-heptane was used as fuel. DL-MCs (1 g) were taped to a glass slide, and the glass was placed 20 cm from the fire. The time until the fire was completely suppressed was recorded, and the temperature was measured to verify the cooling extinguishment. 

## 3. Results and Discussion

### 3.1. Fabrication of Double-Layered Microcapsule (DL-MC)

**Figure 2:**
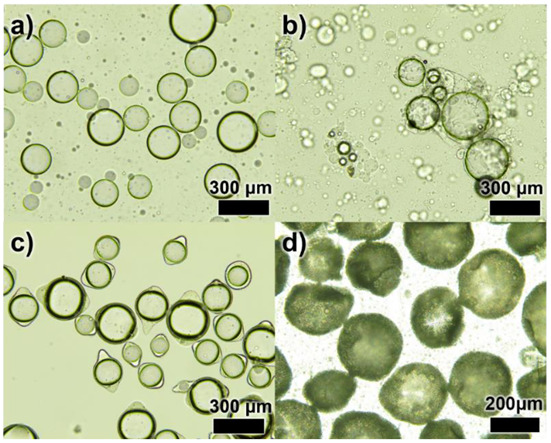
Optical images of the microencapsulation process; (**a**) an emulsification step, (**b**) a complex coacervation step, (**c**) a crosslinking step and (**d**) UF resin coated DL-MCs.

Figure 2 shows the optical microscopic images of each step of DL-MCs preparation. In the emulsion formation step, it was clearly shown that MNFB maintained stable spherical shapes in the O/W emulsion, and GB-SHMP was not observed, as it was fully dissolved in water (Figure 2a and Figure S1). Below pH_ci_, the COO^−^ groups, which had a negative charge, were transformed into neutral COOH groups with H^+^ of aqueous media. Simultaneously, the NH_2_ groups turned into NH_3_^+^ groups, which were positively charged, and then, the negatively charged SHMP molecules in the aqueous solution could interact and agglomerate with the positively charged NH_3_^+^ GB groups, resulting in the formation of tiny transparent coacervate (Figure S1b) [23,24,25,26]. Microencapsulation by coacervate of GB-SHMP can be explained by the phenomenon of Pickering emulsion stabilization. Protein agglomeration acted in the same way, as the particles stabilized the emulsion and were placed on its surface. Yan et al. reported several studies on Pickering emulsion formation by protein aggregates, such as gelatin-pectin [27]. Similarly, the GB-SHMP were adsorbed on the MNFB core surface when the pH values decreased to ca. 4.8 (Figure 2b). The GB-SHMP coacervates on the MNFB surface were cross-linked by glutaric aldehyde as a cross-linking agent, which resulted in the formation of a robust shell (Figure 2c). Subsequently, DL-MCs showed an irregular shell compared to MCs due to the UF resin coating (Figure 2d).

**Figure 3:**
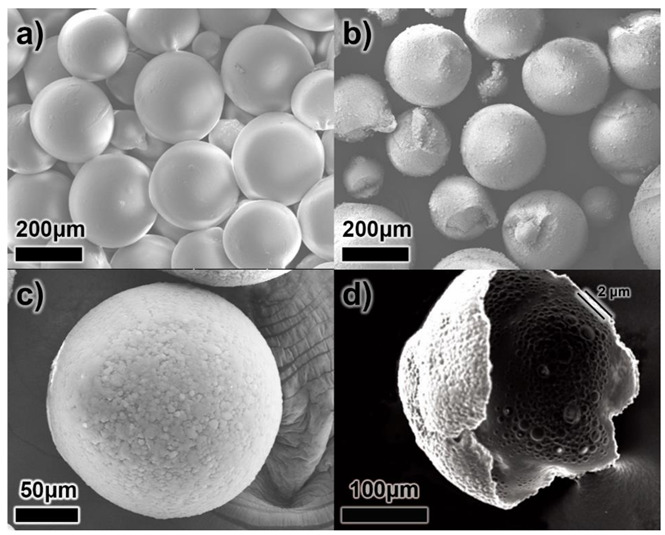
SEM images of microcapsules; (**a**) GB-SHMP microcapsules without UF resin coating (MCs), (**b**,**c**) with UF resin coating (DL-MCs) and (**d**) broken DL-MC.

Figure 3 shows the SEM images of both MCs without UF resin coating (secondary outer shell) and DL-MCs with UF resin coating. As shown in Figure 3a, MCs without UF resin coating have a smooth surface compared to DL-MCs after coating. It could be easily seen that the MCs were connected to each other. On the other hand, the DL-MCs showed a rocky surface of the outer shells without particle aggregation (Figure 3b,c). 

In addition, it was shown that double-layered shell thickness was about 2 μm (Figure 3d). It should be noted that the shell morphology changed slightly after secondary outer shell UF resin coating. As shown in Figure 3b, some DL-MC particles exhibit a deformed spherical shape, while MC showed a perfectly spherical particle (Figure 3a). This is due to the fact that not only did the polymerization/condensation reaction between amine and hydroxyl functional groups occur under acid conditions, but a non-uniform UF resin coating also appeared when the UF resin layer formed on the surface of the inner gelatin shell layer. 

**Figure 4:**
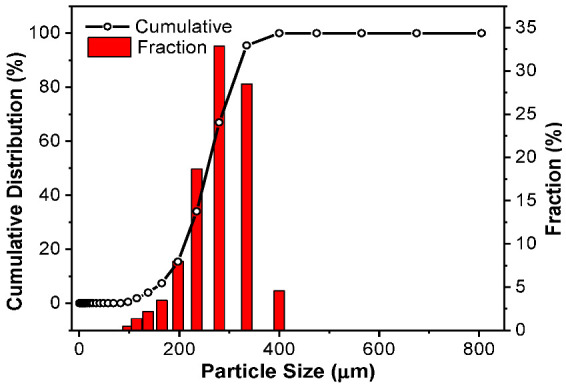
DL-MC particle size distribution.

DL-MC particle size distribution was also investigated by using a particle size analyzer. As shown in Figure 4, the particle showed an almost normal distribution in the range of 100 to 400 μm. The average particle diameter was ca. 309 μm. 

### 3.2. DL-MC Characteristics

**Figure 5:**
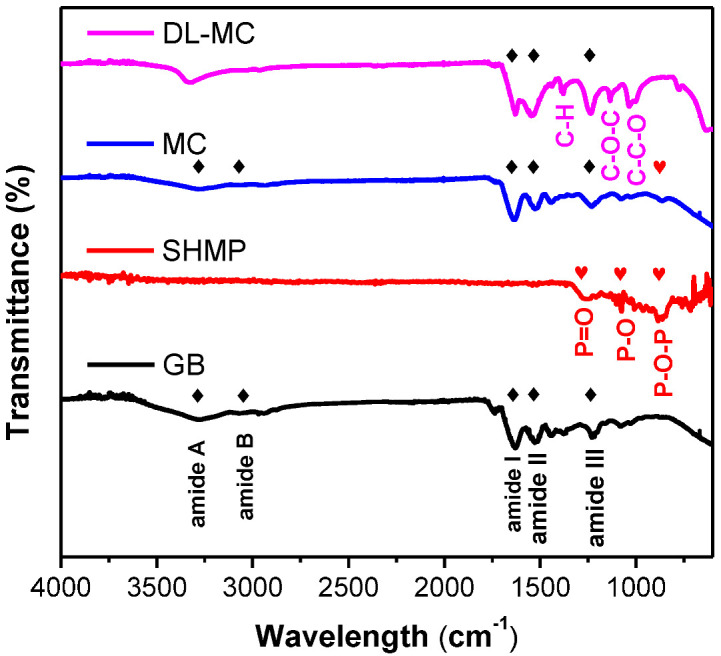
FTIR spectra of microcapsules and microcapsule components (◆: GB, ♥: SHMP).

The surface functional groups of the prepared MC samples (MC and DL-MC) were confirmed by FTIR analysis. The FTIR spectra of GB, SHMP, MC and DL-MC are shown in Figure 5, and their representative characteristic peaks were well observed. GB showed typical vibration peaks corresponding to amide A (3270~3330 cm^−1^), amide B (3070 cm^−1^), amide I (1630 cm^−1^), amide II (1520 cm^−1^) and amide III (1200–1230 cm^−1^), respectively. Both MC and DL-MC showed similar patterns of FTIR spectra with typical peaks related to amide A (3280 cm^−1^), amide B (3070 cm^−1^), amide I (1630 cm^−1^), amide II (1520 cm^−1^) and amide III (1200–1230 cm^−1^), respectively. This indicated that both microcapsule samples had polymer shells successfully fabricated with GB [28,29,30,31]. SHMP showed typical peaks related to vibration of P=O (1240 cm^−1^), P-O (1070 cm^−1^) and 867 cm^−1^ (P-O-P), respectively. The MC sample showed an identical FTIR peak associated with P-O-P vibration at 867 cm^−1^ [32]. However, P-O-P-related vibration was not clearly observed in the DL-MC sample. It is possible that this was attributed to the formation of a relatively thick UF resin layer on the gelatin layer, resulting in a weak related signal SHMP compared to other substances. The DL-MC sample exhibited new peaks corresponding to the asymmetric stretching of C-O-C (1130 cm^−1^), C-C-O and C-H stretching mode of CH_2_OH at 1020 cm^−1^, 1380 cm^−1^, respectively, where they can typically be observed in UF resin [31,33,34,35].

In addition, the amide A peak was slightly shifted from 3280 to 3330 cm^−1^, which can be explained by the hydroxyl group (-OH) generated during the UF resin in situ polymerization [33] and the bonds between the amine and hydroxyl groups of gelatin and UF resin [32]. Thus, it should be concluded that both the inner GB-SHMP shell and the outer UF resin layer were successfully formed by chemical bonding, and the double-layered shell of DL-MC was more robust and had higher strength.

**Figure 6:**
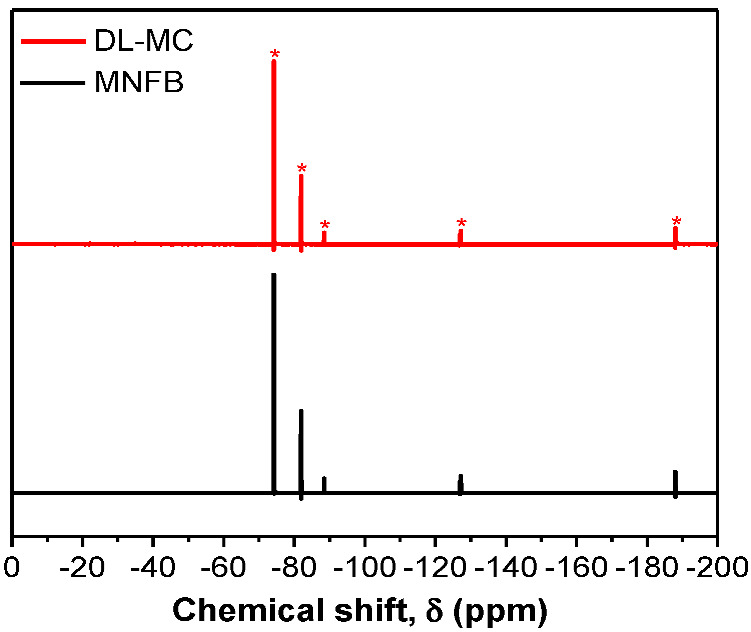
^19^F−NMR spectra of non-flammable liquid and DL-MC.

To verify the presence of MNFB as DL-MC core material, ^19^F-NMR was conducted with acetone-D6 as a solvent, which was similar to the method of Yim et al. [12]. The DL-MCs were placed in a solvent and shattered with a glass rod to extract the MNFB from the DL-MCs. The solvent containing the extracted substances from DL-MCs was filtered. The filtered liquid was charged for NMR analysis and compared with pure MNFB. To ensure the stability of the sample, we used DL-MCs samples, which were aged for 100 days after preparation for this analysis. As shown in Figure 6, the solvent extracted from DL-MCs showed the same NMR peaks as compared to the peaks of pure MNFB. In addition, the relative peak intensity of the DL-MC is completely identical to that of the reference MNFB. This indicates that MNFB is the basic substance in DL-MC and is well maintained as the initial condition for a long time (ca. 100 days) without any change. Based on the above results, it can be concluded that DL-MC has a well-defined core-double shell structure, which consists of a stable MNFB core and both an inner GB-SHMP shell layer and an outer UF resin one. 

### 3.3. Thermal Properties

The thermal properties of microcapsules containing non-flammable agents were the most important factors in fire suppression applications. When a fire occurs, this induces the ambient temperature to rise sharply, as a result of which, the microcapsules release the core of the non-flammable fire suppression agent. The narrow range of operating temperature means that the fire suppression chemicals can be released in the target temperature range, which reduces the probability of device failure [8,9].

**Figure 7:**
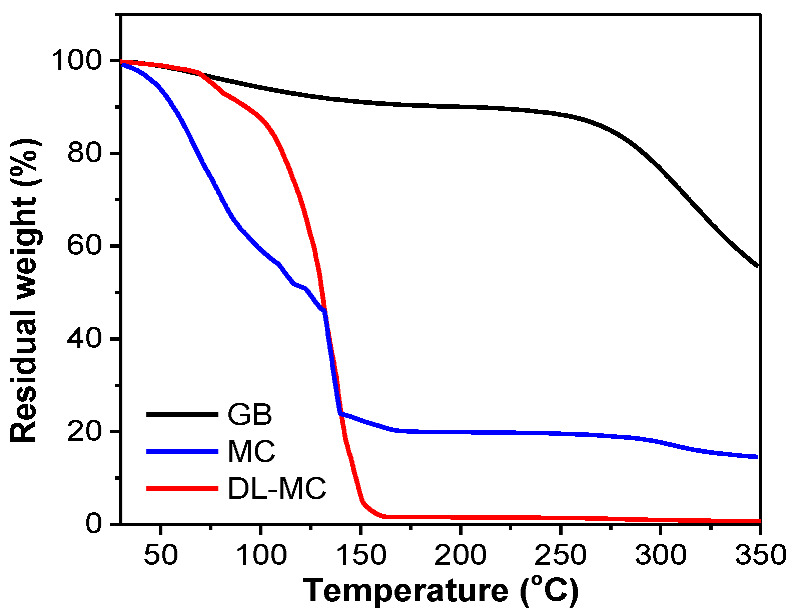
Thermogravimetric analysis (TGA) of GB, MC and DL-MC.

**Figure 8:**
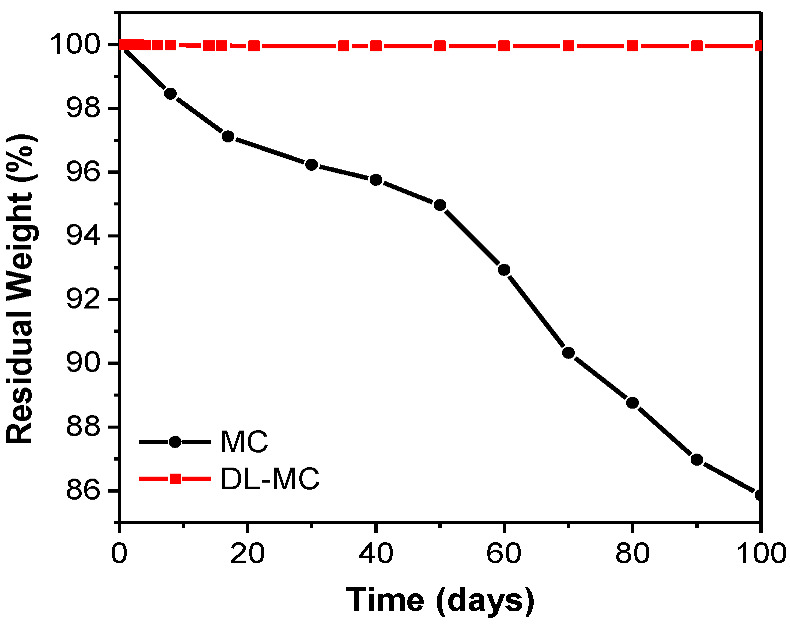
MCs and DL-MCs weight changes under ambient conditions.

Figure 7 shows the TGA curves of the GB, MC and DL-MC samples. Pure GB showed a minor mass loss of up to approx. 250 °C, which is attributed to the evaporation of weakly adsorbed water molecules and impurities. It showed a sequential significant loss above approx. 250 °C, which corresponds to the pyrolysis peak of gelatin alone (Figure 7) [36,37]. The MC exhibits a continuous mass loss in the room temperature range to approx. 130 °C and a dramatic loss in the range of 130 to 140 °C. This indicates that the volatile core materials (MNFB) are continuously vaporized and released from room temperature to 140 °C. In TGA analysis, we can also estimate how long the core substance (MNFB) can be stored for. To obtain TGA results, we used both MC and DL-MC samples after a 100-day stability test. Over 100 days, the core substance, MNFB, in MC is significantly released, while DL-MC shows negligible MNFB loss, as shown in Figure 8. Since the TGA data exhibited the relative percentage of residual mass, although it seems that MC has a large mass of shell residue, it practically has a relatively large portion of shell material with the same absolute mass due to loss of MNFB during the stability test. MC has a large mass ratio of shell residual to core materials after the stability test and the same absolute mass compared to the initial state.

In the TGA results, DL-MC exhibited a minor loss up to about 100 °C and sequential sharp losses ranging from 100 to 150 °C. This means that the core substance (MNFB) is released mainly at a specific temperature range from 100 to 150 °C [8,9,12,13]. The small decrease in DL-MC around 100 °C was attributed to the evaporation of water (ca. 12%). Compared to the mass loss of MC, DL-MC showed a negligible loss below 100 °C and a large loss at specific temperatures. This indicates that DL-MC was more able to prevent the loss of core materials, and the probability of malfunction was also expected to be lower than that of MC. Based on the results, it can be concluded that the DL-MC double-layered shell can play an important role in the stable storage of core materials and the release of fire suppression material at target temperatures (e.g., 100~140 °C) in fire accident situations. It can be explained that the phenomenon is attributed to the formation of the double shell structure. The MC sample should have a tiny pore on the surface after GB layer formation, followed by cross-linking with glutaraldehyde. However, DL-MC may have a thicker UF resin outer shell layer, and the pore space of the inner GB layer should be significantly blocked by the outer UF layer. Thus, it inhibits the loss of internal materials and improves the thermal stability of the microcapsules. As shown in Figure S2, MC showed a broad mass loss below 150 °C, which indicated that the core substance can be easily released if the shell material is not stable. However, DL-MC showed a large peak around 150 °C and relatively small peaks below that temperature. This implied that DL-MC can release the core material, MNFB, in a targeted range of temperatures and have a narrow operation temperature region, which indicated the low malfunction risk in fire suppression.

As shown in Figure S3, the heat flow of DL-MC tended to decrease as the temperature increased below 50 °C. It was responsible for the heat absorption of MNFB, and the strong endothermic peak near 150 °C was induced by the internal MNFB release and vaporization. The narrow range of temperature drop around 150 °C presented in Figure 7 and Figure S3 indicated that the double-layered shell structure stably protects the core materials, and the probability of malfunction should be low for actual fire suppression. A relatively small endothermic peak appearing near 260 °C was due to the pyrolysis of gelatin [37].

In the case of polymer-coated microcapsules for fire suppression applications, it was important to prevent the loss and evaporation of the core material with a low boiling point [8,9]. Furthermore, the high long-term stability can guarantee a reduction in maintenance costs when replacing the microcapsules. Vilesov et al. [9] tried to minimize the number of pores in the shell layer by the addition of montmorillonite and reported that the loss resistance of the core material was dramatically enhanced. A similar tendency was also presented by Bae et al. [21] who investigated the effect of clay content on the barrier properties of fish gelatin film. In this work, we also confirmed that the resistance to the loss of the core substance, MNFB, was significantly improved by fabricating the double-layered shell structure.

We figured out that the core substance stably existed in the double shell for over 100 days, which was confirmed by observation of the weight change of the DL-MC sample for 100 days after preparation. As shown in Figure 8, MC microcapsules with single-layered shells showed about 14% of mass loss; consequently, DL-MC showed only 0.005% of mass loss after 100 days. According to this result, it was confirmed that the long-term stability of the loss of MNFB could be enhanced by UF resin coating on the inner shell of the gelatin.

### 3.4. Fire Suppression Test

**Figure 9:**
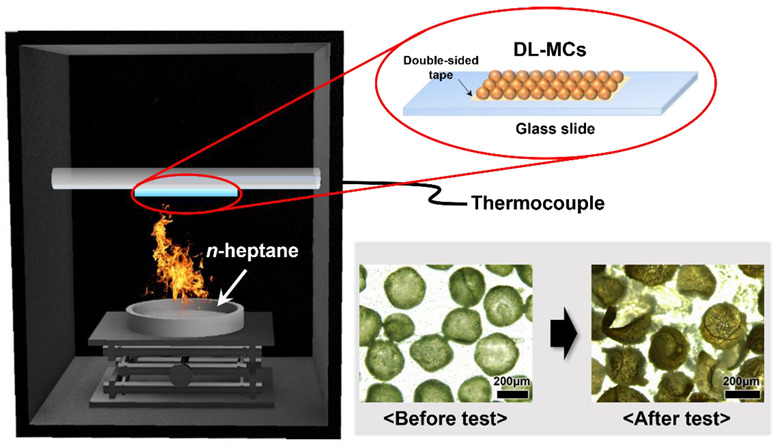
Schematic diagram of the fire suppression test on a laboratory scale.

A laboratory scale fire suppression test for DL-MC was performed in a container shown in Figure 9. Adhesive tape was attached to the glass slide with dried DL-MCs (1 g), and a thermocouple was installed on the back of the glass slide. N-heptane (50 mL) was used as the fuel, and the fire suppression test was started by ignition. Figure 9 shows that DL-MCs were deformed after the test due to the release of the core substance, MNFB.

**Figure 10:**
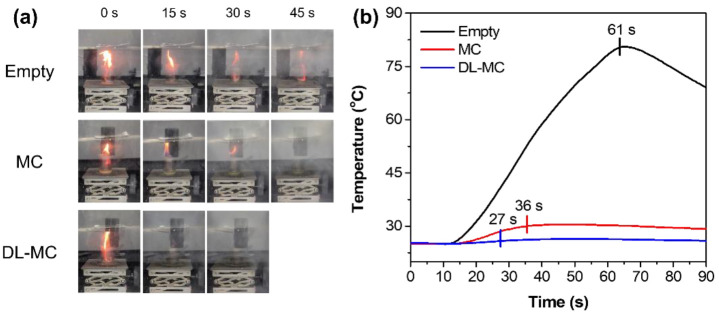
Laboratory scale fire suppression test; (**a**) Digital images of the fire suppression test, (**b**) Temperature deviation in the laboratory scale fire suppression test.

Digital images of a laboratory scale fire suppression test with time intervals (every 15 s) and profiles of temperature changes are shown in Figure 10a,b, respectively. In the case of an empty plate, the fire was extinguished naturally after 61 s because all the fuel had been exhausted. As shown in Figure 10b, the temperature increased drastically in less than 61 s and then gradually decreased due to the removal of the heat source. When we used the fire suppression microcapsules, complete extinction toward fire was achieved in 36 s and 27 s for MC and DL-MC, respectively. The fire flame was exposed to microcapsule samples, and it was possible to increase the local temperature. Then, as the outer shell was torn, the internal extinguishing agent was released to suppress the fire. It was confirmed that the range of the temperature rise was small compared to the empty plate because the MNFB was vaporized and simultaneously absorbed the surrounding heat. In particular, when DL-MC was applied as a fire suppression agent, it could be seen that the fire flame was almost extinguished for about 15 s. This should be because DL-MC contains more extinguishing agents and has longer storage performance compared to MC. As shown in Figure 10b, more internal substance can be released in this way, and more MFNB is evaporated, resulting in more heat absorbed and negligible temperature changes. Therefore, DL-MC proved to be more effective in initial fire suppression compared to the single-layered microcapsule samples.

## 4. Conclusions

Double-layered microcapsules were prepared by additional coating with a UF resin layer on GB-SHMP-based single-layered microcapsules fabricated by a complex coacervation method. Based on the characterization results, we confirmed that the UF resin layer and GB-SHMP layer were strongly bonded together, which improved properties such as thermal durability and long-term stability of the microcapsules. In addition, the synthesized double-layered microcapsule (DL-MC) sample exhibited high content of the fire suppression agent as the core substance and stable thermal properties, and the fire suppression performance was significantly improved compared to the single-layered microcapsule. DL-MCs containing MNFB were proven to have excellent cooling extinguishment advantages, which are short suppression times and little temperature change in laboratory scale fire suppression tests. It is believed that our microcapsules with a double-layered structure can significantly reduce the probability of a fire appliance malfunction and can act as a method of suppressing the effects of electric fire accidents in energy storage systems.

## Figures and Tables

**Table 1:** Physicochemical properties of core materials, methoxynonafluorobutane (MNFB).

	Molecular Weight [g/mol]	Boiling Point [°C]	Freeze Point [°C]	Liquid Density [g/mL]	Vapor Pressure [mmHg]	Ozone Depletion Potential
MNFB	250	61	−135	1.52	202	0.00

## Data Availability

Not applicable.

## Supplementary Materials

The following supporting information can be downloaded at: https://www.mdpi.com/article/10.3390/ma15217831/s1, Figure S1: Turbidimetric analysis for optimization of microencapsulation; (a–d) optical images according to pH value in the turbidimetric analysis graph; Figure S2: DTG data of GB, MC and DL-MC; Figure S3. Different scanning calorimeter (DSC) of DL-MC.
