# Supplementary material for: Double-Layered Polymer Microcapsule Containing Non-Flammable Agent for Initial Fire Suppression
Source: Materials (Basel). 2022 Nov 6;15(21):7831. doi: 10.3390/ma15217831 (PMC9659137; doi:10.3390/ma15217831)
Supplement: Supplementary file 1 [file materials-15-07831-s001.zip › materials-1960920-SI.pdf]

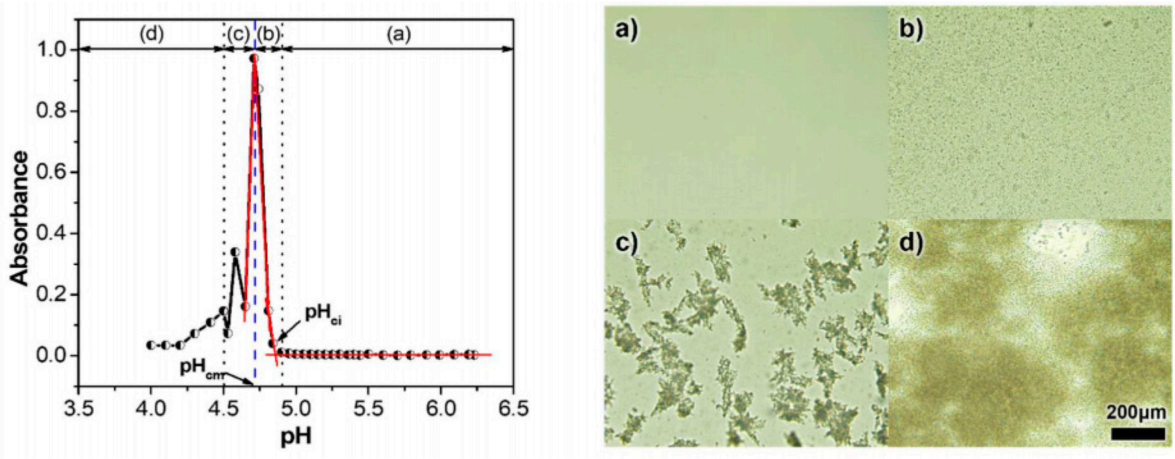

Figure S1. Turbidimetric analysis for optimization of microencapsulation; **a**–**d**).optical images according to pH value in the turbidimetric analysis graph.

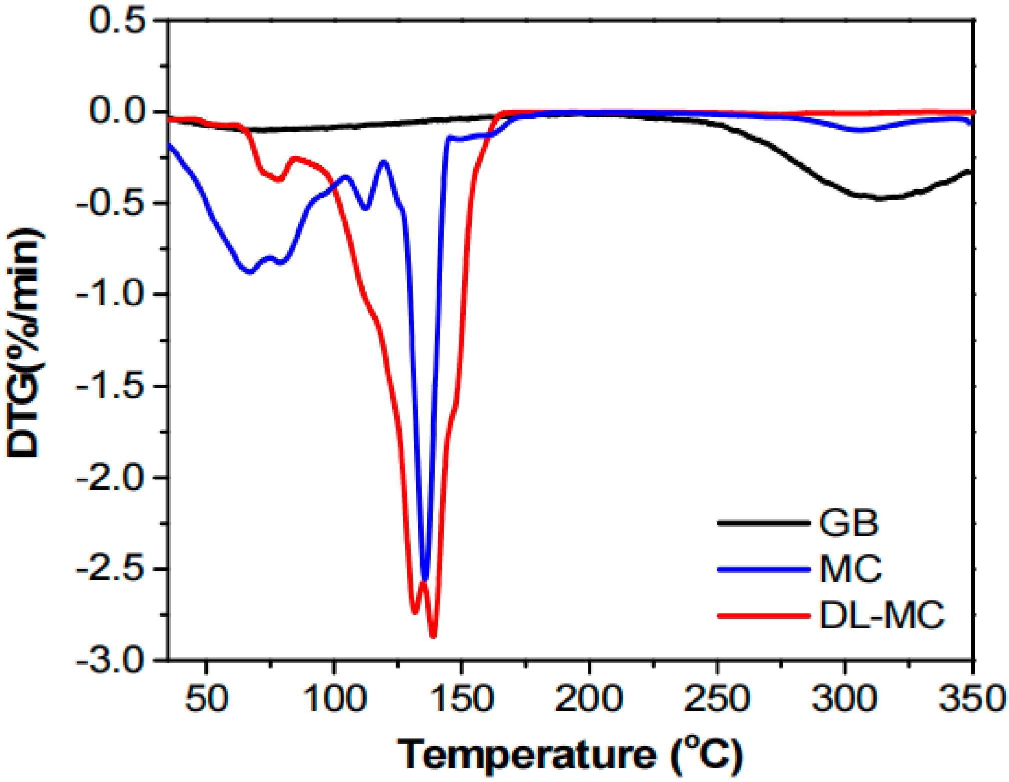

Figure S2. DTG data of GB, MC and DL-MC.

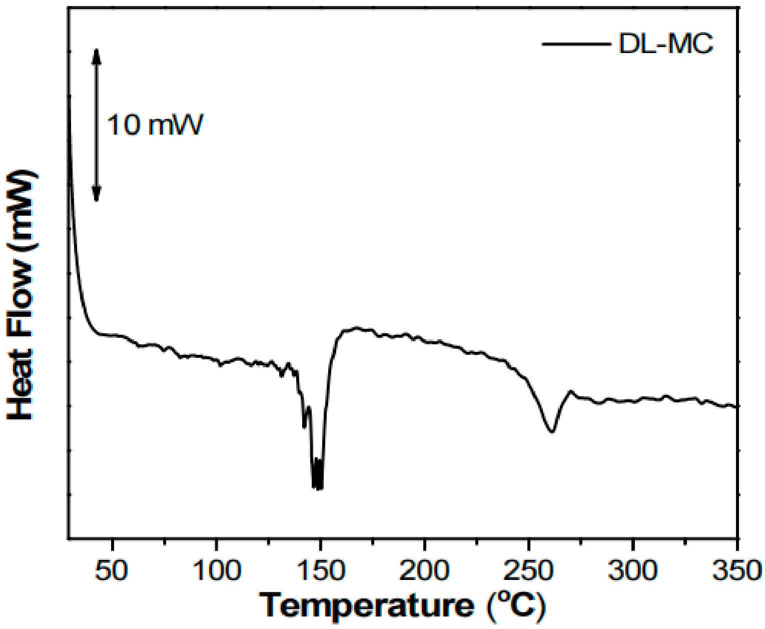

Figure S3. Different scanning calorimeter (DSC) of DL-MC.
